# miRNAs and lncRNAs in tomato: Roles in biotic and abiotic stress responses

**DOI:** 10.3389/fpls.2022.1094459

**Published:** 2023-01-11

**Authors:** Qian Li, Heng Shen, Shoujuan Yuan, Xigang Dai, Changxian Yang

**Affiliations:** ^1^ Key Laboratory of Horticultural Plant Biology, Ministry of Education, Huazhong Agricultural University, Wuhan, China; ^2^ School of Life Sciences, Jianghan University/Hubei Engineering Research Center for Protection and Utilization of Special Biological Resources in the Hanjiang River Basin, Wuhan, China; ^3^ Hubei Hongshan Laboratory, Huazhong Agricultural University, Wuhan, China

**Keywords:** tomato, miRNA, lncRNA, biotic and abiotic stresses, regulatory network

## Abstract

Plants are continuously exposed to various biotic and abiotic stresses in the natural environment. To cope with these stresses, they have evolved a multitude of defenses mechanisms. With the rapid development of genome sequencing technologies, a large number of non-coding RNA (ncRNAs) have been identified in tomato, like microRNAs (miRNAs) and long non-coding RNAs (lncRNAs). Recently, more and more evidence indicates that many ncRNAs are involved in plant response to biotic and abiotic stresses in tomato. In this review, we summarize recent updates on the regulatory roles of ncRNAs in tomato abiotic/biotic responses, including abiotic (high temperature, drought, cold, salinization, etc.) and biotic (bacteria, fungi, viruses, insects, etc.) stresses. Understanding the molecular mechanisms mediated by ncRNAs in response to these stresses will help us to clarify the future directions for ncRNA research and resistance breeding in tomato.

## Introduction

Tomato (*Solanum lycopersicum*) is one of the most important vegetable crops in the world. The Food and Agriculture Organization of the United Nations estimated that fresh tomato production is close to approximate 190 million tonnes in 2020. However, tomato production is under threat due to various abiotic and biotic stresses, including abiotic stresses: drought, salinization, high temperature, cold, nutrient deficiencies, etc; biotic stresses: insects, fungi, bacteria, nematodes, viruses, etc. Besides protein-coding genes, a large number of non-coding RNAs (ncRNAs) have been recently identified under various stress conditions in tomato, such as 70 miRNAs induced by *Phytophthora infestans* infection, 790 miRNAs in pollen under heat stress conditions, and 23 lncRNAs induced after inoculated with *Ralstonia solanacearum* (Luan et al., 2015; Keller et al., 2020; Cao et al., 2022). These results demonstrate that miRNAs and lncRNAs possibly play critical roles in tomato defense against abiotic and biotic stresses.

Based on their transcript length and function, ncRNAs can be divided into several different types, including microRNA (miRNA), long non-coding RNA (lncRNA), small interfering RNA (siRNA), circular RNA (circRNA), piwi-interacting RNA (piRNA) (Zhang et al., 2019). Because ncRNAs play important roles in the responses to various stresses, mining and characterizing more stress-related ncRNAs will provide new strategies for tomato breeding against abiotic and biotic stresses in the future (Wang et al., 2015). In this review, we mainly summarize the research progress on the ncRNAs (miRNAs and lncRNAs) biogenesis and their regulatory roles in the responses to biotic and abiotic stresses in tomato.

## Transcription, processing, and transportation of miRNA

In plants, most miRNAs are transcribed by RNA polymerase II (pol II) from MIR genes or intronic regions into primary miRNAs (pri-miRNAs) with imperfect stem-loop structures (Lee et al., 2004; Cai et al., 2004; Jones-Rhoades et al., 2006). The HYL1-SE-DCL1 complex plays vital roles in miRNA processing. In the nucleus, the stem-loop region of pri-miRNA can be recognized by dsRNA-binding protein HYL1, which assists RNase III endonuclease DCL1 to precisely cut pri-miRNA. Meanwhile, the C2H2 zinc finger protein SE was reported to help DCL1 to cut accurately and help to activate the activity of DCL1 (Yang et al., 2006; Dong et al., 2008; Fang et al., 2015). 2 bases will protrude at the 3’ end after two-step cleavage of DCL1 (Khvorova et al., 2003; Song et al., 2007; Li and Yu, 2021). In order to protect miRNAs from degradation, the methyltransferase HUA ENHANCER 1 (HEN1) will methylate the 2’-OH position of the 3’-end to increase the stability of double-stranded miRNAs (Yu et al., 2005; Zhang et al., 2022).

In plants, AGO1 is the master protein of RNA-induced silencing complex (RISC) and plays vital roles in most miRNA-mediated transcriptional gene silencing (TGS) and post-transcriptional gene silencing (PTGS). AGO1 has two domains, PAZ and PIWI, which are closely related to RNAi in plants (Cerutti et al., 2000; Hammond et al., 2000; Song et al., 2003; Baumberger and Baulcombe, 2005). In addition, The chaperone protein HSP90 can help AGO1 perform spatial folding to change the conformation of proteins to accommodate the correct localization and binding of double-stranded miRNAs (Iki et al., 2010).

The latest research showed that there are two ways for the assembly of RISC. One is that the processed miRNA/miRNA* duplex is first transported into the cytoplasm through HASTY (HST), where the assembly of RISC is carried out in the cytoplasm, and the guide strand (miRNA) guides the duplex to be loaded on the AGO1 protein (Krista et al., 2003). The thermodynamic stability of the 5’ terminal determines the degradation of one of the double-stranded RNA. Studies showed that the double-stranded RNA-binding protein DRB1 and phosphatase CPL assist the RISC in selecting single-stranded RNA with low thermal stability of 5’ terminal (Eamens et al., 2009; Manavella et al., 2012). The miRNA* are degraded at this point, completing the assembly. This assembly method is mainly aimed at the degradation or translation inhibition of mRNA on the endoplasmic reticulum. The other is that the miRNA/miRNA* duplex is directly guided to the AGO1 protein in the nucleus for RISC assembly, and the miRNA* is degraded at the same time (Bologna et al., 2018). After the assembly is completed, it is transported to the cytoplasm by EXPO1, and then downstream regulates the expression of downstream mRNAs (Bologna et al., 2018). Due to the limited space of this paper, it is not detailed here. Recent studies on miRNA biogenesis have been summarized in detail by Song et al. (Song et al., 2019).

What’s more, many studies have shown that the processing and biogenesis of miRNAs are regulated by the external environment, which means that plants can adapt to changes in the external environment by inhibiting or activating the production of miRNAs (for details, see Manavella et al., 2019).

## Classification and action mechanisms of lncRNAs

In recent years, a large number of lncRNAs have been identified in plants due to the continuous development of high-throughput sequencing, which are longer than 200 nt in length and have no protein-coding ability (Liu et al., 2012). Based on their relative position to the protein-coding gene, lncRNAs were roughly divided into including long intergenic non-coding RNA (lincRNA), intronic lncRNA (incRNA), sense lncRNA, and antisense lncRNA (St. Laurent et al., 2015; Ma et al., 2013). Growing evidence showed that lncRNAs primarily interact with DNA, mRNA, protein, and miRNA, and consequently play crucial roles at the epigenetic, transcriptional, post-transcriptional, translational, and post-translational levels (Kung et al., 2013; Lucero et al., 2021). For example, an intronic long noncoding RNA (COLDAIR) in Arabidopsis binds to polycomb repressive complex 2 (PRC2) and helps PRC2 localize to the FLOWERING LOCUS C (FLC) under cold conditions. Then, PRC2’s master-acting component histone methyltransferase mediates histone H3 Lys^27^ trimethylation (H3K27me3). H3K27me3 is enriched on FLC chromatin during vernalization, leading to stable epigenetic repression of flowering in Arabidopsis (Heo and Sung, 2011).

LncRNAs also act as enhancer RNAs, which enriches RNA polymerase II to gene promoters, thereby promoting gene transcription (Kim et al., 2010). Besides, some lncRNAs can act as endogenous target mimics (eTMs) of miRNAs to compete with mRNAs for binding to miRNAs, thereby alleviating the inhibitory effect of miRNAs. Under phosphate deficiency condition, miRNA399 in Arabidopsis directs the cleaving of mRNAs encoding ubiquitin-binding enzymes and activate the content of Pi in buds, while lncRNA IPS1 binds to miR399 and inhibits the cleavage effect of miRNA399 on the target mRNA (Franco-Zorrilla et al., 2007). Studies have shown that some lncRNAs can also be processed by different Dicer-like enzymes to produce miRNAs, siRNAs, and tasiRNAs, and some of them can be induced by water stress, salt stress, and phosphate starvation stress (Ben et al., 2009). lncRNA has become the focus of research because of its various regulatory functions in plants, and the complex mechanism of action of lncRNA in plants has been summarized by some articles (for details, see Wierzbicki et al., 2021; Lucero et al., 2021; Jampala et al., 2021). A thorough understanding of the role of lncRNA in plants will help provide some ideas for the study of tomato lncRNA, and accelerate the use of lncRNA to cultivate tomato crops with strong adaptability to extreme environments.

## The role of miRNAs in abiotic stresses in tomato

As an important participant in the stress regulatory network, miRNAs respond to abiotic stresses by inhibiting the expression of their downstream mRNAs (Ma et al., 2022). In recent years, abiotic stress responsive miRNAs have been gradually identified in tomato (**Supplementary Table 1**), but their functions remains largely unknown (Cardoso et al., 2018). In this section, we mainly focus on the regulatory roles of miRNAs under various abiotic stresses in tomato, including drought, low/high temperature, salinization, and nutrient deficiency.

### Drought stress

Drought is one of important limiting factors that limits plant growth, development, yield, and quality. Plants have evolved a variety of complex mechanisms to adapt to drought stress, like ABA signaling pathway. A whole bunch of miRNAs that respond to drought stress have been identified. For example, 688 miRNAs were found to be differentially expressed in different tissues of two tomato cultivars with different drought tolerances (Candar-Cakir et al., 2016). Among them, miR160, miR165, miR166, miR171, and miR9552, etc., may be involved in tomato response to drought stress. These miRNAs target glycosyltransferases and dehydrated reactive proteins, thereby regulating stomatal motility or root development in response to drought (Candar-Cakir et al., 2016). Interestingly, miR9552 specifically expressed in roots was obviously up-regulated in tolerant varieties but down-regulated in sensitive varieties. The prediction indicated that UDP-glucosyltransferase is one of the targets of miR9552. Studies have shown that this enzyme can regulate the biosynthesis of some secondary metabolites (flavonoids) and plant hormones (IAA, ABA) to regulate the sensitivity of plants to drought (Candar-Cakir et al., 2016; Dong et al., 2020).

**Figure 1:**
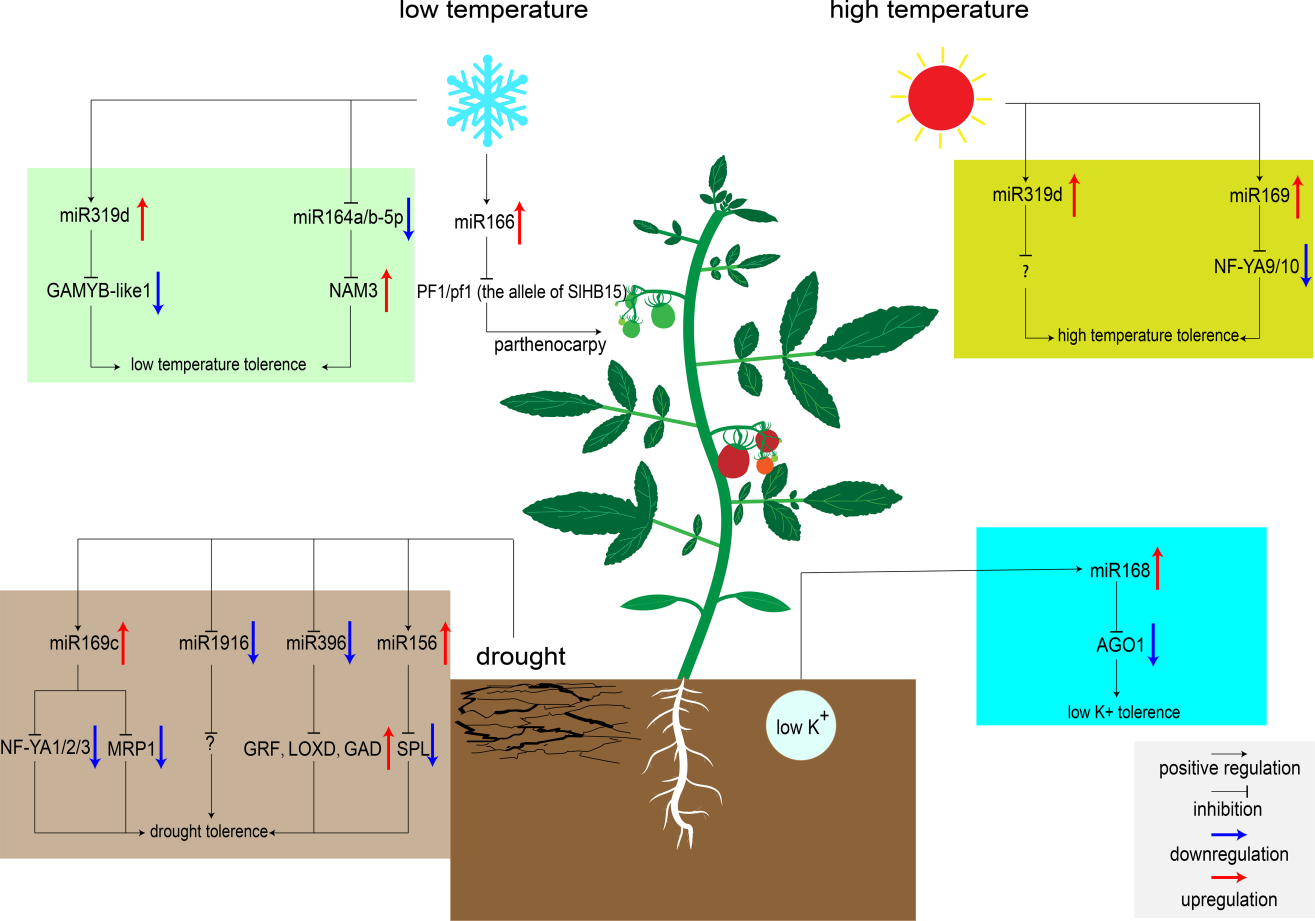
MiRNAs regulate tomato response to abiotic stresses. MiR319d was induced by low temperature to inhibit the expression of target genes *GAMYB-like1* and improving tomato cold tolerance (Shi et al., 2019). MiR166 was also induced to express by low temperature. When embryonic development is blocked, low temperature induces parthenocarpy in heterozygous genotype plants (Clepet et al., 2021). Low temperature will down-regulate the expression of miR164a/b-5p, release the inhibition of NAM3, and improve the cold tolerance of tomato (Dong et al., 2022). High temperature upregulates the expression of miR319d and miR169, and inhibiting the transcription of downstream target genes to improve tomato heat tolerance (Shi et al., 2019; Rao et al., 2022). However, the target genes of miR319d at high temperature are unknown. miR169c and miR156 were up-regulated under drought, inhibited the expression of downstream transcription factors, and improved tomato tolerance to drought (Zhang et al., 2011; Visentin et al., 2020). While miR1916 and miR396 were down-regulated under drought condition, regulating downstream signaling pathways to improve tomato tolerance to drought (Chen et al., 2019; Fracasso et al., 2021). The target genes of miR1916 under drought stress are currently unknown. Low potassium stress induces the expression of miR168, inhibits the expression of AGO1, and enhances K^+^ uptake by regulating downstream signaling pathways (Liu et al., 2020).

After treated with drought stress, 24 conserved miRNAs differentially expressed in M82, and 43 conserved miRNAs differentially expressed in introgression line 9 (IL-9) have been identified. These miRNAs target some protein kinases, oxidoreductases, drought stress proteins, and several transcription factors (Liu et al., 2017). These miRNAs in tomato may respond to drought stress by regulating the expression levels of these genes to alter reactive oxygen species (ROS) signaling pathways, hormone signaling pathways, and osmotic stress signaling pathways *in vivo* (Liu et al., 2017). Over-expression of miR169c reduces the opening of stomata and slows down the transpiration rate (Li et al., 2008). Four targets of miR169c, NF-YA1/2/3 and SlMRP1 (ABC transporter), were down-regulated in the transgenic tomato plants over-expressing miR169c compared to wild type (**Figure 1**). Among them, NF-YA has been reported to play an important role in Arabidopsis drought response (Li et al., 2008). MRP was associated with ion channel activity and stomatal motility (Klein et al., 2004; Zhang et al., 2011). Recently, miR396 was found to be transcriptionally down-regulated under drought stress in *S. pennellii*. Measurement of physiological indicators demonstrated that the density and size of stomata were inhibited in the miR396 down-regulated plant in comparison to WT, in which the transpiration efficiency and water dispersion loss were reduced (Fracasso et al., 2021). In particular, miR396 can also improve tomato water use efficiency by regulating the GABA metabolic pathway. On the one hand, GABA can induce the decrease of tomato stomatal conductance. On the other hand, GABA can enhance the antioxidant capacity of cells and reduce the damage caused by ROS (Wang et al., 2017; Fracasso et al., 2021). Additionally, a recent study showed that silencing of miR1916 increases drought tolerance in tomato and tobacco, which targets histone deacetylases (HDAC) and strictosidine synthase (STR) in tobacco (Chen et al., 2019). These two enzymes can regulate the content of soluble sugar and proline, maintain a balanced osmotic pressure under drought stress, and improve water use efficiency (Chen et al., 2019).

### Low/high temperature stress

Low temperatures can delay tomato development and cause fruit malformation (Barrero-Gil et al., 2016). Exploring low temperature signaling pathway is of great significance to improve the cold tolerance of tomato plants. Recently, hundreds of miRNAs involved in low temperature stress responses have been identified. The majority target genes of these miRNAs encode protein kinases, antioxidant enzymes, and the primary components of the cell wall (Cao et al., 2014). Thus, these genes may be involved in regulating ROS signaling pathways to reduce the cell damage caused by low temperature. Over-expression of miR319d can improve cold tolerance in tomato by regulating ROS signaling pathway. Under low temperature condition, miR319d increased antioxidant enzyme activity by inhibiting cold response factors GAMYB-like1, thereby reducing ROS production and protecting plants from oxidative stress (Shi et al., 2019). In addition, low temperatures also affect tomato fruit setting. New research showed that tomato *SlHB15* is critical for ovule development. When PF1/pf1 (the allele of SlHB15) heterozygous, the cold-inducing factor miR166 can lead to parthenocarpic fruit set under low temperature by regulating the expression of *SlHB15* (Clepet et al., 2021).

In some areas, high temperatures severely limit the tomato yield. Recently, Shi et al. found that miR319d induced the expression of heat-responsive genes *HSP90* and *HsfA1s* under heat stress, which was accompanied by the expression of antioxidant enzymes (Shi et al., 2019). Over-expression of miR319 can enhance the activity of antioxidant enzymes to reduce cell damage caused by ROS under high temperature condition (Shi et al., 2019). Besides this miRNA, miR169 also appears to be involved in heat tolerance regulatory network in tomato. Over-expression of miR169 can significantly inhibit the expression of its target gene NF-YA9/10, and result in the markedly increased activity of its downstream ascorbate peroxidase (APX) (H_2_O_2_ scavenging enzyme), then improved tomato heat tolerance (Rao et al., 2020; Rao et al., 2022). In addition, miR167a acts as a responsive medium under a variety of abiotic stresses. MiR167a was down-regulated after drought, high temperature, and low temperature treatment. Analysis of the miR167a promoter sequence revealed the presence of multiple stress-related cis-acting elements in this region, suggesting that miR167a may be involved in multiple abiotic stress regulatory networks (Jodder et al., 2018). In-depth verification of the function of miR167 may be very beneficial to improve the overall resistance of tomato in the future.

### Salt stress

Salinization reduces plant growth and crop productivity by regulating osmotic pressure, ion exchange, material transport in tomato (Guo et al., 2022). MiRNAs have been found to play crucial regulatory roles in tomato resistance to salt stress. Recently, 145 and 53 differentially expressed miRNAs were identified in *M82* and *S. pennellii* treated with salt, respectively (Wang et al., 2021). PsRNATarget prediction indicated that these miRNAs target some salt stress responsive genes such as MYB, bHLH, WRKY, MADS-box, and TCP transcription factors (Feng et al., 2013; Xu et al., 2021; Zhang et al., 2022). Particularly, laccase enzymes have been reported to be the target of miR397 in Arabidopsis and *Oryza sativa*, which catalyzes phenolic compounds to reduce oxygen to water (Jeon et al., 2012). Tomato plants may also escape from salt stress through the similar process. Moreover, another study showed that the expression level of miR398 gradually decreased from 0 to 24h after treatment with salt, which targets *CSD* (ROS scavenger) and *PR5* (related to biotic and abiotic stresses) (Luan et al., 2014). These results demonstrate that miR398 may be involved in the tomato salt stress regulatory network.

### Nutrient deficiency

Phosphate (P) is one of the nutrients essential for plant growth. In case of P deficiency, tomatoes establish symbiotic relationships with microorganisms such as *arbuscular mycorrhizal* (AM) to cope with P deficiency. Interestingly, miRNAs may also play a vital role in this symbiotic relationship (Smith et al., 2003). By simulating P deficiency and AM inoculation conditions, the researchers found that there were a large number of differentially expressed miRNAs in roots. Among them, miR319, miR394, miR399, miR158 and miR862-3p were differentially expressed in roots under P deficiency and AM symbiotic conditions (Gu et al., 2010). Target prediction showed that most of them are phosphate transporters, aquaporins, and protein kinases (Gu et al., 2010). MiRNAs may enhance the selective uptake of P by tomato by targeting these proteins, and also participate in the exchange of P between AM and plants in response to phosphate stress. Potassium (K^+^) is also an indispensable nutrient of crops, which is indispensable for tomato fruit setting in the late growth period. Potassium deficiency induces changes in many signaling pathways, such as the biosynthesis of ROS and plant hormones, and other miRNA-regulated signaling pathways (Shin and Schachtman, 2004). Over-expression of miR168 can improve tomato tolerance to low potassium stress. Several differentially expressed miRNAs (miR171a, miR530, miR384, miR858, and miR8007b) were identified by high-throughput sequencing of 35S:miR168a, 35S:AGO1, and WT plants (Liu et al., 2020). GO analysis showed that these miRNAs directly regulate the changes of Ca^2+^ signaling pathways and ABA/CTK signaling pathways. Under low potassium stress, miR168 that targets AGO1 regulates downstream signaling pathways to alter root architecture or root sensitivity to K^+^, thereby enhancing K^+^ uptake by root hairs (Xian et al., 2014; Liu et al., 2020).

## The role of lncRNAs in response to abiotic stresses

In recent years, lncRNAs have been identified to be associated with abiotic stresses (Jha et al., 2020). However, the role of lncRNA defense against abiotic stress still remains elusive in tomato. Recently, 1411 lncRNAs have been identified in tomato fruits, of which 239 lncRNAs were differentially expressed under low temperature stress (Wang et al., 2018b). GO and KEGG pathway analysis indicated that the targets of most of these lncRNAs encode chilling-related enzymes, pectinase, membrane lipid peroxidase, etc. In addition, some targets belong to low-temperature-induced WRKY transcription factors and cold responsive genes (Wang et al., 2018b). Moreover, lncRNAs also appear to be involved in salt tolerance in tomato. A total of 133 lncRNAs were up-regulated in *M82* roots, whereas 100 lncRNAs were down-regulated in *S. pennellii* roots after treated with salt (Li et al., 2022). The possible targets of the majority of these lncRNAs were enriched with genes encoding cell wall components, biosynthesis of various amino acids and secondary metabolites, and redox processes (Li et al., 2022). Future functional validation of these lncRNAs will contribute to our understanding the regulatory network underlying salt tolerance in tomato.

Severe changes in ambient temperature and humidity caused cracked tomato fruit. Bacteria can thus easily infect the cracked fruits, then shorten the shelf life of tomato fruits. Changes of cellulose, hemicellulose, and some pectin, are considered as major factors in fruit cracking (Khadivi-Khub, 2015). Through transcriptome sequencing, 2509 lncRNAs were identified at different time points after saturated irrigation of two tomatoes with different fruit cracking resistance (Xue et al., 2020). The targets mRNAs of these differentially expressed lncRNAs encode pectinases, hydrolases and ethylene response factors. It is well known that ethylene plays an important role in promoting fruit ripening and softening, which can induce the expression of some genes related to cell wall (such as cell wall degrading enzyme TAPG1) and then lead to cell wall degradation (Xue et al., 2020). In addition, lncRNA1459 has been reported to be involved in the regulation of tomato fruit ripening. The content of ethylene was lower in the fruits of the *lncRNA1459* mutant at ripening stages than that in wild type (Li et al., 2018). LncRNA1459 may also regulate tomato fruit cracking through the ethylene signaling pathway. Functional identification and pathway analysis of the lncRNAs that respond significantly in the dehiscence transcriptome data will help improve the shelf life of tomato without affecting its flavor.

## The role of miRNAs in tomato response to biotic stresses

Biotic stresses, including bacteria, fungi, viruses, insects, and others, result in the loss of important crop plants globally. Nowadays, pathogen-associated molecular patterns (PAMP)-triggered immunity (PTI) and effector-triggered immunity (ETI), also refereed as horizontal resistance and vertical resistance, activate defense reponses after detect invading pathogens. For the first one, the surface-localized pattern recognition receptor (PRR) recognizes pathogens and trigger PAMP of immune responses (PTI), which is generally considered as the first layer of defense in plants. This defense response is generally accompanied by stomatal closure, Ca^2+^ signaling, production of ROS and protein kinases, and biosynthesis of jasmonate/salicylic acid to limit pathogen invasion (Takahashi et al., 2011; Pombo et al., 2014; Lal et al., 2018; Liu et al., 2019; Yuan et al., 2021). In addition, the intracellular nucleotide-binding site and leucine-rich repeat receptors (NBS-LRR), also known as R-resistant proteins, could detect effector proteins from pathogens and induce ETI. This immune response is accompanied by hypersensitive reaction (HR), which leads to programmed cell death (Chisholm et al., 2006). In most cases, the cascade of PTI and ETI regulates downstream signaling networks and induces resistance proteins to protect plants against infection from various pathogens (Qi et al., 2011).

In the last decades, researchers mainly focus on the transcriptional regulation during tomato immunity, like disease-resistant QTLs or resistance genes. However, a large proportion of ncRNAs also made many contributions in defense against pathogens. A number of ncRNAs could be induced by biotic stresses, which activate defenses against pathogens by regulating their downstream immune signaling pathways (**Supplementary Table 2**).

**Figure 2:**
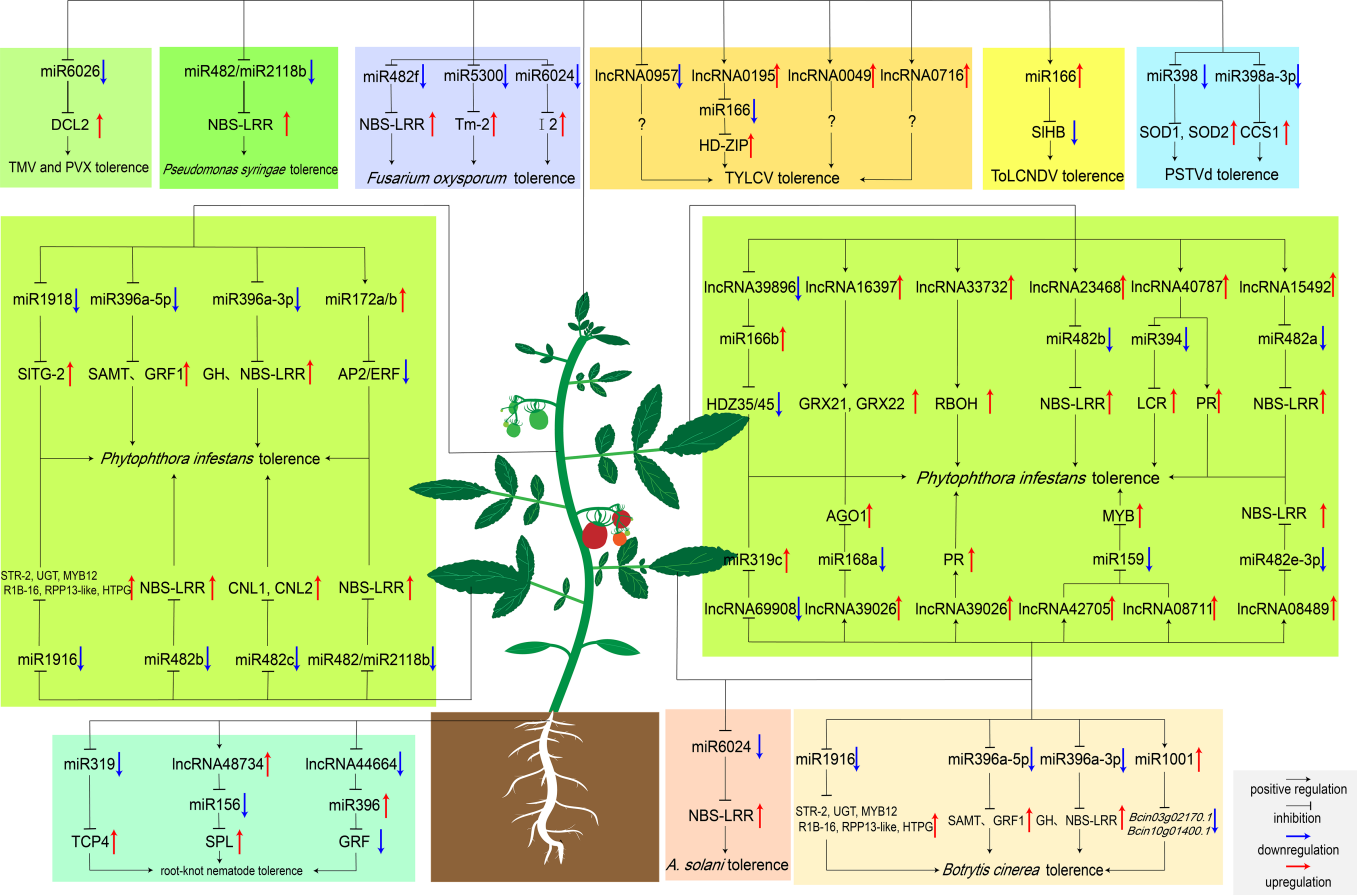
MiRNAs and lncRNAs regulate tomato response to biotic stresses. Boxes with different colors represent pathogen species regulated by ncRNAs. Particularly, *Bcin03g02170.1* and *Bcin10g01400.1* encode the ATP-dependent metallopeptidase and cysteine-type endopeptidase in *B cinerea*, respectively. MiR1001 reduces the virulence of *B cinerea* by repressing the expression of these two genes (Meng et al., 2020).

MiRNAs have been revealed to play essential roles in tomato immune regulatory network, some of which respond to pathogen invasion by regulating the expression of R genes (**Figure 2**). MiR482 and miR2118 function as potential regulators that induce immune defense against the invasion of pathogens in tomato. Among them, miR482 targets the disease resistance protein genes and induces RNA polymerase VI to produce secondary siRNA. When pathogen invasion, miR482-mediated RNA silencing was inhibited and defense protein genes were gradually expressed to halt viral and bacterial invasion (Shivaprasad et al., 2012). Increasing evidence showed that the miR482 family members may negatively regulate tomato resistance to pathogens. Recently, silencing miR482b with a short tandem target mimic (STTM) resulted in increased resistance of tomatoes to *P. infestans*. Four targets (*Solyc02g036270, Solyc04g009070, Solyc12g016220*, and *Solyc05g008070*) have been identified by degradome sequencing, all of which were NBS-LRR resistance genes and up-regulated after silencing miR482b (Jiang et al., 2018). Furthermore, silencing miR482c by STTM induced up-regulation of the expression levels of two R genes with coiled-coil domains, which enhanced tomato resistance to late blight (Hong et al., 2019). Besides, miR6022 and miR6024 were also predicted to regulate tomato resistance to pathogens by targeting the R genes *Hcr9* and *Tm-2*, respectively (Li et al., 2012). Moreover, the *I2* gene is essential for tomato resistance to *Fusarium oxysporum.* MiR6024 can target *I2* and strictly maintains the transcription level of *I2* to prevent pathogen invasion (Wei et al., 2014).

A large amount of plant viruses form anti-defense strategies in the process of continuous evolution. This process induces the expression of some miRNAs. After infection, cucumber mosaic virus and tomato aspermy virus produce viral proteins that interact with the AGO1 protein to disrupt the cleavage of the AGO1 (Feng et al., 2009). The expression levels of miR159, miR162, miR168 and miR171 changed significantly to varying degrees (Feng et al., 2009). In addition, tomato bushy stunt virus (TBSV) also produces a PTGS repressor protein P19 after invasion in tomato, which is able to bind to the miRNA/miRNA* duplex to inhibit the assembly of the RISC (Chapman et al., 2004). The researchers found that the activities of miR160a and miR164 were significantly inhibited through simulating TBSV invasion by highly expressing the P19 protein (Stav et al., 2010). Among them, miR164 targets *GOBLET*, which regulates the initiation of secondary lobule and apical meristem. Therefore, the P19 protein disrupts the cleavage ability of miR164 and leads to round leaves, rolled leaves, and restricted development in tomato (Yael et al., 2009). In addition, the transcriptional-activator protein AC2, the replication enhancer protein AC4, and the pre-coat protein AV2 encoded by the tomato leaf curl new Delhi virus inhibit the RNAi pathway and also induce the expression of some miRNAs in the process (Van et al., 2002). Microarray analysis showed that the expression of miR159, miR172 and miR319 was up-regulated 3-5 times, increased with the infection time (Naqvi et al., 2010). Other miRNAs such as miR162, miR168, miR396, miR397, miR398, miR408, and miR447 were also up-regulated after infection, but miR160, miR169, miR170, and miR391 were down-regulated (Naqvi et al., 2010). The necrotizing bacterium *Botrytis cinerea Pers. Fri.* can also secrete some toxic compounds when invading plants. After *B. cinerea* inoculation, miR159, miR169, miR319, miR394, miR1919, and miRn1 were significantly up-regulated, whereas miR160 and miR5300 were significantly down-regulated. Moreover, TCP, F-BOX proteins and pathogenesis-related transcription factors were targeted by miR319, miR394 and miRn1, respectively (Jin and Wu, 2015). These results suggest that miR319, miR394 and miRn1 may be involved in the regulatory network of tomato resistance to *B. cinerea.* These miRNAs can be used as biomarkers to observe virus invasion, which can also be used to develop antiviral strategies (Naqvi et al., 2010). Complete characterization and annotation of the signaling pathways regulated by these miRNAs will help reveal the mechanism of virus invasion, so that we can propose disease-resistant strategies.

## The role of lncRNAs in tomato response to biotic stresses

In recent years, lncRNAs have been reported to play critical regulatory roles in defense against biotic stresses (Zaynab et al., 2018). The process of plant-pathogen interaction induces some lncRNAs significant changed, and in-depth study of these significantly changed lncRNAs may promote their use as biomarkers of virus invasion (Prasad and Prasad, 2021). *Pseudomonas syringae pv. tomato* (*Pst*), injects effector proteins into tomato upon invasion. These effector proteins disrupt the PTI response and induce ETI, which is also accompanied with significant responses of some lncRNAs. At different time periods after *Pst* invaded, a large number of differentially expressed lncRNAs have been identified in tomato leaves. These lncRNAs were involved in the regulation photosynthesis and the expression of genes encoding cell wall kinases and glutathione S-transferases (GST) after the induction of ETI and PTI (Rosli et al., 2021). These transcriptome data facilitated our characterization of lncRNA regulatory modules related to *Pst* resistance in addition to protein-coding genes.

Late blight, which is mainly caused by the *P. infestans*, is a very harmful disease in tomato production. By comparing transcriptome data on *P. infestans* resistance and susceptibility tomato varieties, 1037 differentially expressed genes and 688 differentially expressed lncRNAs have been identified. GO and colocalization network showed that 56 differentially expressed genes encode oxidoreductases (Cui et al., 2017). When tomato plants senses *P. infestans* invasion, it rapidly accumulates ROS content to cope with pathogen invasion. At the later stage of infection, some transcription factors such as glutaredoxins (GRXs) reduce cell membrane damage by limiting oxidative stress and scavenging excess accumulated reactive oxygen species (ROS) (Laporte et al., 2012). After *P. infestans* invasion, tomato limited the accumulation of ROS *in vivo* through the lncRNA16397-SlGRX22 module to reduce the cell membrane damage (Cui et al., 2017). In addition, WRKY transcription factors can improve tomato biotic and abiotic stresses tolerence. Recently, Cui et al. found that the promoter of lncRNA33732 was recognized and activated by tomato WRKY 1. The activated lncRNA33732 can positively regulate the expression of respiratory burst oxidase (RBOH) to increase H_2_O_2_ content to protect against early infection by *P. infestans* (Cui et al., 2019).

Tomato yellow leaf curl virus (TYLCV), transmitted by *Bemisia tabaci*, is one of the most severe and destructive viruses in the world. After infected with TYLCV, the young tissues turned yellow, the leaves curled upward, and the development was delayed, and the yield was greatly reduced (Glick et al., 2009). A large number of differentially expressed lncRNAs have been identified in tomato after artificial inoculation with TYLCV. RT-PCR results showed that lncRNA0850, lncRNA1167, lncRNA0372, lncRNA1514, lncRNA0667, lncRNA0957, lncRNA0519 and lncRNA0494 responded significantly. After silencing lncRNA0957 through VIGS, it was found that the symptoms of transgenic tomato plants were significantly reduced (Wang et al., 2018c). These markedly expressed lncRNAs may be involved in tomato defense against TYLCV networks. Many lncRNAs related to pathogen resistance have not been characterized. Making full use of various sequencing data for functional verification of lncRNAs in response to various pathogens is conducive to the development of disease-resistant tomato from a new perspective.

## NcRNAs regulate hormone synthesis in response to biotic and abiotic stresses in tomato

Plant hormones such as ABA, IBA, JA, and GA are key factors in tomato development and in response to biotic and abiotic stresses (Bari and Jones, 2009). Increasing evidence demonstrate that ncRNAs can regulate various phytohormone biosynthetic pathways in response to biotic and abiotic stresses in tomato. *B. cinerea* and *P. infestans* infection of tomato induced downregulation of miR396a-3p/5p expression, and promoted the synthesis of salicylic acid carboxymethyltransferase, glycosyl hydrolase and the expression of R genes (Chen et al., 2017). In this process, tomato plants also prevent the invasion of pathogens by altering SA and JA biosynthetic signals (Chen et al., 2017). In addition, miR1127-3p was significantly inhibited after attacked by *B. cinerea*, its target WRKY75 was increased by more than 70 times, and the content of JA was significantly increased in tomato. This confirmed that miR1127-3p improved the resistance to *B. cinerea* by inducing the expression of *WRKY75* and then regulating the downstream JA signaling pathway (López-Galiano et al., 2018). Previous studies showed that miR482b can negatively regulate resistance to *P. infestans* by targeting NBS-LRR genes. In particular, GO and KEGG pathway indicated that over-expression of miR482b also down-regulated the expression of genes related to α-linolenic acid, cysteine and methionine metabolic pathways. These genes are crucial component in regulating MAPK signaling pathway, JA and ET biosynthesis (Jiang et al., 2019).

Biotic and abiotic stresses have been reported to tightly associated with ethylene (Achard et al., 2006; Achard et al., 2007). Ethylene response factor (ERF) acts as a signaling factor at the end of the ethylene signaling pathway, and is involved in resistance to some fungal invasions and resistance to osmotic stress (Pirrello et al., 2006; Feng et al., 2020). 397 lncRNAs have been identified by deep sequencing of *sense-LeERF1* and *antisense-LeERF1* transgenic tomato. These lncRNAs were predicted to target some ethylene signaling related genes like F-BOX proteins, auxin response factors, cytochrome proteins, and MADS-box transcription factors (Wang et al., 2018a). Additionally, miRNAs could assist tomato escaping from low temperature by regulating the ethylene signaling pathway. The latest study showed that tomato miR164a/b-5p targets the downstream transcription factor NAM3 after cold signal transmitted from the cell membrane into the cell. NAM3 regulates the ethylene synthesis pathway and increases ethylene content. In addition, NAM3 directly regulates cold response genes (COR47like) to improve tomato cold tolerance (Dong et al., 2022). Moreover, miR1917 negatively regulates the ethylene response key inhibitor *CONSTITUTIVE TRIPLE RESPONSE4* to regulate ethylene content in tomato, suggesting that the miR1917-mediated ethylene synthesis pathway is likely involved in other stress-regulating networks (Wang et al., 2018d).

ABA has been also demonstrated to be associated in regulating the expression of stresses-related genes. The application of exogenous ABA also caused significant changes in some stressed-related ncRNAs in tomato. Recently, 269 markedly expressed miRNAs have been identified by transcriptome sequencing of after treated with exogenous ABA in tomato. RT-PCR results showed that miR6024-3p, miR7997a, miR172a, miR5658, miR5301, miR169b, miR159 and miR165a-3p were up-regulated, but miR7997c, novel_miR_392, novel_miR_191 and miR171d were down-regulated (Cheng et al., 2016). Moreover, these miRNAs target several defense genes such as NBS-LRR, AP2/EREBP, and several genes associated with plant stress tolerance like MYB, bZIP, bHLH, and NAC (Cheng et al., 2016). Furthermore, ABA and strigolactone can jointly regulate tomato tolerance to drought. The latest study showed that under drought condition, miR156 requires strigolactone induction and regulates stomatal movement through regulation of ABA signaling pathway (Visentin et al., 2020). Interestingly, miR156-OE can reduce stomatal conductance and lower free ABA content *in vivo* compared with WT, in contrast to the conclusion that ABA induces stomatal closure (Visentin et al., 2020). It has been speculated that the reduced stomatal conductance may be due to the increased sensitivity of plants to ABA by overexpression of miR156 (Visentin et al., 2020). ABA and JA can also coordinately regulate stomatal movement in response to drought stress, and the miRNAs in tomato appear to be involved in this signaling network. A recent study showed that under drought stress, miR396 was down-regulated to activate the biosynthesis of JA and ABA (Fracasso et al., 2021). The activation of the JA pathway could increase the content of JA-Ile *in vivo*, which was conducive to protecting chloroplasts and photosignaling systems. In addition, down-regulation of miR396 expression also reduced stomatal density and size by maintaining balance of JA and ABA hormone levels (Fracasso et al., 2021). NcRNAs regulate a wide range of plant hormone signaling pathways. It is necessary to explore and identify more hormone-related ncRNAs to accelerate our understanding of the hormone signaling network in response to biotic and abiotic stresses in tomato. Not only that, exogenous hormones can also induce significant changes in some stress-related ncRNAs in tomato to regulate downstream signals, which makes it possible to use exogenous hormones to induce the expression of some stress-related ncRNAs in the future to improve tomato resistance to certain diseases. Absolutely, this all builds on our mature understanding of ncRNA regulatory networks.

## MiRNA and lncRNA interactions in tomatoes respond to biotic stress

In order to cope with the complex and variable natural environment, different ncRNAs often cross-talk to regulate molecular pathways. Early studies in Arabidopsis have shown that lncRNAs can act as a target for miRNAs to inhibit the miRNA-mediated mRNAs cleavage (Wu et al., 2013). This pattern of regulating downstream gene expression after transcription also exist in tomato. For example, degradome sequencing predicts that lncRNA42705/lncRNA08711, lncRNA39896, lncRNA11265/lncRNA15816 competitively bind to miR159, miR166b, and miR164-5P, and regulate the expression of downstream MYB, NAC and HD-ZiP genes, respectively. These lncRNAs-miRNAs may form the regulatory network of tomatoes against *P. infestans* (Cui et al., 2020). On top of that, silencing of lncRNA42705 and lncRNA08711 by VIGS can obviously up-regulate the expression of miR159, resulting in a decrease in the expression of the MYB gene. Compared to WT, VIGS-silenced transgenic tomato plants had more severe disease (Cui et al., 2020). Additionally, lncRNA08489 was able to compete to bind miR482e-3p as an eTM. Sequence analysis showed that there were lncRNA08489 binding sites at the ninth and tenth nucleotides of the 5 ‘terminal of miR482e-3p. When infected with *P. infestans*, lncRNA08489 can bind to miR482e-3p and inhibit its expression, and the expression levels of downstream resistance genes gradually increase to improve the tolerance of tomato to pathogens (Liu et al., 2022). Another study of lncRNA-miRNA interactions during *P. infestans* invasion showed that lncRNA40787 can act as an competitive endogenous RNA (ceRNA) for miR394. Over-expression of lncRNA40787 can significantly reduce the expression level of miR394, inhibit the cleavage effect of miR394 on the JA biosynthetic gene LCR, increase JA biosynthesis, and enhance tomato resistance to *P. infestans* (Zhang et al., 2021). In terms of resisting the invasion of *P. infestans*, there is also a feedback regulation between lncRNA15492 and miR482b, and they balance the expression of R gene in different periods to resist pathogen invasion and reduce cell damage. After *P. infestans* infected tomato, lncRNA15492 inhibited the expression of miR482a precursor, some NBS-LRRs were thus gradually activated and expressed, improving the resistance to *P. infestans* in tomato (Jiang et al., 2020). When the accumulation of NBS-LRR in tomato reaches a certain level, miR482a will in turn cleave lncRNA15492 to relieve the inhibition of miR482a, resulting in the accumulation of mature miR482a and reducing the expression of resistance genes (L Jiang et al., 2020).

After inoculation with TYLCV, the expression levels of lncRNA0195 and lncRNA1077 increased significantly in tomato. It was predicted that lncRNA0195 and lncRNA1077 could serve as “bait” for miR166 and miR399, respectively (Wang et al., 2015). Silencing lncRNA0195 by VIGS found that the accumulation of virus in transgenic plants was more than 70 times that of control plants. Particularly, the expression level of miR166 target gene III HD-Zip decreased. This proves that lncRNA0195 can indeed competitively bind to miR166 to regulate tomato resistance to TYLCV (Wang et al., 2015).

Some bacteria are also able to induce the expression of lncRNAs in tomato, which can act as “bait” to induce some miRNAs to mediate defense against pests. When the commercial biocontrol bacterium Sneb821 is inoculated into *moneymaker*, it induces the expression of lncRNA48734 and lncRNA44664. The former negatively regulates miR156 to activate the transcription of SPL genes, and to accumulate the ROS content in the roots to resist the invasion of *root-knot nematodes* (Yang et al., 2020). The latter negatively regulates miR396 to inhibit GRFs, reduce the size of syncytids, thereby inhibiting the nutrient source of *root-knot nematodes* and limiting their development (Hewezi et al., 2012; Yang et al., 2020).

The mechanism of this lncRNA acting as eTM to regulate the level of miRNA to regulate downstream signaling pathways is widely present in tomato. Establishing and enriching the lncRNAs-miRNAs network will help accelerate our understanding of the interaction between tomato ncRNAs and biotic stresses, and provide a molecular basis for future molecular breeding to obtain highly resistant tomatoes.

## Prospect and conclusion

The rapid development of high-throughput multi-omics techniques has become increasingly sophisticated in identifying ncRNAs that respond to different stresses. Most stress-related ncRNAs are characterized and annotated in Arabidopsis and legumes (Chand et al., 2021). However, the role of the majority of stress-related ncRNAs in tomatoes still remains uncharacterized. New transgenic technologies, such as artificial miRNAs (amiRNAs), STTMs, and CRISPR-Cas9, has been successfully used to verify the function of miRNA and lncRNA, and in the future, single gene editing of certain ncRNAs can be used to enhance good agronomic trait (Yogindran and Rajam, 2021). At present, a large number of ncRNAs have been identified in tomato under different stress conditions, but their targets and regulatory pathways are rarely fully characterized. Functional characterization and mechanism analysis of these ncRNAs with significant stress response will facilitate the selection of resistant varieties in the future. Meanwhile, in the era of whole genome sequencing, more and more reference genomes of different species make it easier to analyze the conservatism and sequence similarity of ncRNAs between species. The role and molecular mechanism of ncRNAs in response to stresses in tomato as one of model plants, especially miRNAs and lncRNAs, help us to reveal the function of stress-related ncRNAs in other species. In the future, various biotic and abiotic stresses will occur frequently, which seriously threaten tomato yield (Ashish et al., 2022). Single-gene resistance gradually failed to meet crop production in some areas, strategies for developing resistant crops in new directions are urgently needed. Therefore, unfolding the secrets of ncRNAs responsive to different stresses will provide new strategies for tomato resistance breeding against biotic and abiotic stresses, and also insights for improving the adaptability of other vegetable crops against adverse stresses.

## Author contributions

QL, HS, and SY conceived the idea. QL wrote the manuscript. QL, HS, SY, CY, and XD revised and edited the manuscript. All authors agree to the final version of the manuscript.
