# Supplementary material for: miRNAs and lncRNAs in tomato: Roles in biotic and abiotic stress responses
Source: Front Plant Sci. 2023 Jan 11;13:1094459. doi: 10.3389/fpls.2022.1094459 (PMC9875070; doi:10.3389/fpls.2022.1094459)
Supplement: Supplementary file 1 [file Table_1.docx]

**Supplementary table 1.** Tomato miRNAs that resist to abiotic stresses and their targets.

| Abiotic stresses miRNAs targets References |
| --- |
| Drought miR169c *NF-YA1/2/3、SlMRP1* (Zhang et al., 2011)  miR1916 (Chen et al., 2019a)  miR396  *GRF、LOXD、GAD* (Fracasso et al., 2021)  miR156 *SPL* (Visentin et al., 2020)  Cold stress miR164a/b-5p *NAM3* (Dong et al., 2022)  miR319d *GAMYB-like1* (Shi et al., 2019)  miR166 *HB15A* (Clepet et al., 2021)  Heat stress miR319d (Shi et al., 2019)  miR169 *NF-YA9/10* (Rao et al., 2022)  potassium deficiency miR168 *AGO1A* (Liu et al., 2020) |

**Reference**

Zhang, X., Zou, Z., Gong, P. et al. (2011) Over-expression of microRNA169 confers enhanced drought tolerance to tomato. Biotechnol Lett. 33, 403–409.

Chen, L., Meng, J., Luan, Y. (2019a) miR1916 plays a role as a negative regulator in drought stress resistance in tomato and tobacco. Biochem Biophys Res Commun. 508(2):597-602.

Fracasso, A., Vallino, M., Staropoli, A., Vinale, F., Amaducci, S., Carra, A. (2021) Increased water use efficiency in miR396-downregulated tomato plants. Plant Sci. 303:110729.

Visentin, I., Pagliarani, C., Deva, E., Caracci, A., Turečková, V., Novák, O., Lovisolo, C., Schubert, A., Cardinale, F. (2020) A novel strigolactone-miR156 module controls stomatal behaviour during drought recovery. Plant Cell Environ. 43(7):1613-1624.

Dong, Y., Tang, M., Huang, Z., Song, J., Xu, J., Ahammed, G.J., Yu, J., Zhou, Y. (2022) The miR164a-NAM3 module confers cold tolerance by inducing ethylene production in tomato. Plant J. 111(2):440-456.

Shi, X., Jiang, F., Wen, J., Wu, Z. (2019) Overexpression of Solanum habrochaites microRNA319d (sha-miR319d) confers chilling and heat stress tolerance in tomato (S. lycopersicum). BMC Plant Biol. 19(1):214.

Clepet, C., Devani, R.S., Boumlik, R., Hao, Y., Morin, H., Marcel, F., Verdenaud, M., Mania, B., Brisou, G., Citerne, S., Mouille, G., Lepeltier, J.C., Koussevitzky, S., Boualem, A., Bendahmane, A. (2021) The miR166-SlHB15A regulatory module controls ovule development and parthenocarpic fruit set under adverse temperatures in tomato. Mol Plant. 14(7):1185-1198.

Rao, S., Gupta, A., Bansal, C., Sorin, C., Crespi, M., Mathur, S. (2022) A conserved HSF:miR169:NF-YA loop involved in tomato and Arabidopsis heat stress tolerance. Plant J. 112(1):7-26.

Liu, X., Tan, C., Cheng, X., Zhao, X., Li, T., Jiang, J. (2020) miR168 targets Argonaute1A mediated miRNAs regulation pathways in response to potassium deficiency stress in tomato. BMC Plant Biol. 20(1):477.
