# Supplementary material for: miRNAs and lncRNAs in tomato: Roles in biotic and abiotic stress responses
Source: Front Plant Sci. 2023 Jan 11;13:1094459. doi: 10.3389/fpls.2022.1094459 (PMC9875070; doi:10.3389/fpls.2022.1094459)
Supplement: Supplementary file 2 [file Table_2.docx]

**Supplementary table 2**. Tomato ncRNAs that resist to biotic stresses and their targets.

| Biotic stresses ncRNAs targets/modules References |
| --- |
| *Tobacco mosaic virus* miR6026 *DCL2* (Wang et al., 2018d)  *potato virus X* miR6026 *DCL2* (Wang et al., 2018d)  *Pseudomonas syringae* miR482/miR2118b *NBS-LRR* (Canto-Pastor et al., 2019)  *Fusarium oxysporum* miR482f *NBS-LRR* (Ouyang et al., 2014)  miR5300 *Tm-2* (Ouyang et al., 2014)  miR6024 *I2* (Wei et al., 2014)  *P. infestans* miR1918 *SlTG-2* (Luan et al., 2016)  miR396a-5p/-3p *NBS-LRR、SAMT、GRF1、GH* (Chen et al., 2017)  miR172a/b *AP2、ERF* (Luan et al., 2018)  miR1916 *STR-2、UGT、MYB12、*  *R1B-16、RPP13-like* and *HTPG* (Chen et al 2019b)  miR482c *SlCNL1、SlCNL2* (Hong et al., 2019)  miR482b *NBS-LRR*  (Jiang et al., 2018)  miR482/miR2118b *NBS-LRR* (Canto-Pastor et al., 2019)  lncRNA16397 *SlGRX22* (Cui et al., 2017)  lncRNA33732 *RBOH* (Cui et al., 2019)  lncRNA15492 miR482a-NBS-LRR (Jiang et al., 2020)  lncRNA08489 miR482e-3p-NBS-LRR (Liu et al., 2022)  lncRNA23468 miR482b-NBS-LRR (Jiang et al., 2019a)  lncRNA40787 miR394-*LCR、PR*  (Zhang et al., 2021)  lncRNA39026 miR168a-*AGO1*、*PR*  (Hou et al., 2020)  lncRNA42705、lncRNA08711 miR159-*MYB* (Cui et al., 2020)  lncRNA39896 miR166b-*HDZ34/45* (Hong et al., 2022)  lncRNA69908 miR319c (Zhou et al., 2022)  *Botrytis* *cinerea*  miR1916 *STR-2、UGT、MYB12、*  *R1B-16、RPP13-like* and *HTPG* (Chen et al., 2019b)  miR396a-5p/-3p *NBS-LRR、SAMT、GRF1、GH* (Chen et al., 2017)  miR1001 *Bcin03g02170.1、Bcin10g01400.1* (Meng et al., 2020)  *root-knot nematode* miR319 *TCP4* (Zhao et al., 2015)  lncRNA48734 miR156-*SPL* (Yang et al., 2020)  lncRNA44664 miR396-*GRFs* (Yang et al., 2020)  *Tomato Yellow Leaf Curl Virus* lncRNA0957 (Wang et al., 2018b)  lncRNA0049、lncRNA0716 (Wang et al., 2015)  lncRNA0195 miR166-HD-ZIP (Wang et al., 2015)  *Potato Spindle Tuber Viroid* miR398、miR398a-3p  *SOD1、SOD2, CCS1* (Suzuki et al., 2019)  *Tomato leaf curl New Delhi virus* miR166 *HB* (Prasad et al., 2022)  *Alternaria solani* miR6024 *NBS-LRR* (Dey et al., 2022) |

**Reference**

Wang, Z., Hardcastle, T.J., Canto, Pastor. A., Yip, W.H., Tang, S., Baulcombe, D.C. (2018d) A novel DCL2-dependent miRNA pathway in tomato affects susceptibility to RNA viruses. Genes Dev. 32(17-18):1155-1160.

Canto-Pastor, A., Santos, BAMC., Valli, A.A., Summers, W., Schornack, S., Baulcombe, D.C. (2019) Enhanced resistance to bacterial and oomycete pathogens by short tandem target mimic RNAs in tomato. Proc Natl Acad Sci U S A. 116(7):2755-2760.

Ouyang, S., Park, G., Atamian, H.S., Han, C.S., Stajich, J.E., Kaloshian, I., Borkovich, K.A. (2014) MicroRNAs suppress NB domain genes in tomato that confer resistance to Fusarium oxysporum. PLoS Pathog. 10(10):e1004464.

Wei, C., Kuang, H., Li, F., Chen, J. (2014) The I2 resistance gene homologues in Solanum have complex evolutionary patterns and are targeted by miRNAs. BMC Genomics. 15(1):743.

Luan, Y., Cui, J., Wang, W., Meng, J. (2016) MiR1918 enhances tomato sensitivity to Phytophthora infestans infection. Sci Rep. 6:35858.

Chen, L., Meng, J., Zhai, J., Xu, P., Luan, Y. (2017) MicroRNA396a-5p and -3p induce tomato disease susceptibility by suppressing target genes and upregulating salicylic acid. Plant Sci. 265:177-187.

Luan, Y., Cui, J., Li, J., Jiang, N., Liu, P., Meng, J. (2018) Effective enhancement of resistance to Phytophthora infestans by overexpression of miR172a and b in Solanum lycopersicum. Planta. 247(1):127-138.

Chen, L., Meng, J., He, X.L., Zhang, M., Luan, Y.S. (2019b) Solanum lycopersicum microRNA1916 targets multiple target genes and negatively regulates the immune response in tomato. Plant Cell Environ. 42(4):1393-1407.

Hong, Yu-Hui., Jun, Meng, Xiao-Li, He., Yuan-Yuan, Zhang., and Yu-Shi, Luan. (2019). "Overexpression of MiR482c in Tomato Induces Enhanced Susceptibility to Late Blight" Cells. 8, no. 8: 822.

Jiang, N., Meng, J., Cui, J., Sun, G., Luan, Y. (2018) Function identification of miR482b, a negative regulator during tomato resistance to Phytophthora infestans. Hortic Res. 5:9.

Cui, J., Luan, Y., Jiang, N., Bao, H., Meng, J. (2017) Comparative transcriptome analysis between resistant and susceptible tomato allows the identification of lncRNA16397 conferring resistance to Phytophthora infestans by co-expressing glutaredoxin. Plant J. 89(3):577-589.

Cui, J., Jiang, N., Meng, J., Yang, G., Liu, W., Zhou, X., Ma, N., Hou, X., Luan, Y. (2019) LncRNA33732-respiratory burst oxidase module associated with WRKY1 in tomato- Phytophthora infestans interactions. Plant J. 97(5):933-946.

Jiang, N., Cui, J., Hou, X., Yang, G., Xiao, Y., Han, L., Meng, J., Luan, Y. (2020) Sl-lncRNA15492 interacts with Sl-miR482a and affects Solanum lycopersicum immunity against Phytophthora infestans. Plant J. 103(4):1561-1574.

Liu, W., Cui, J., Luan, Y. (2022) Overexpression of lncRNA08489 enhances tomato immunity against Phytophthora infestans by decoying miR482e-3p. Biochem Biophys Res Commun. 587:36-41.

Jiang, N., Cui, J., Shi, Y., Yang, G., Zhou, X., Hou, X., Meng, J., Luan, Y. (2019a) Tomato lncRNA23468 functions as a competing endogenous RNA to modulate NBS-LRR genes by decoying miR482b in the tomato-Phytophthora infestans interaction. Hortic Res. 6:28.

Zhang, Y.Y., Hong, Y.H., Liu, Y.R. et al. (2021) Function identification of miR394 in tomato resistance to Phytophthora infestans. Plant Cell Rep 40, 1831–1844.

Hou, X., Cui, J., Liu, W., Jiang, N., Zhou, X., Qi, H., Meng, J., Luan, Y. (2020) LncRNA39026 Enhances Tomato Resistance to Phytophthora infestans by Decoying miR168a and Inducing PR Gene Expression. Phytopathology. 110(4):873-880.

Cui, J., Jiang, N., Hou, X., Wu, S., Zhang, Q., Meng, J., Luan, Y. (2020) Genome-Wide Identification of lncRNAs and Analysis of ceRNA Networks During Tomato Resistance to Phytophthora infestans. Phytopathology. 110(2):456-464.

Hong, Y., Zhang, Y., Cui, J., Meng, J., Chen, Y., Zhang, C., Yang, J., Luan, Y. (2022) The lncRNA39896-miR166b-HDZs module affects tomato resistance to Phytophthora infestans. J Integr Plant Biol. 64(10):1979-1993.

Zhou, X., Cui, J., Luan, Y. (2022) Characterization of lncRNAs in mycorrhizal tomato and elucidation of the role of lncRNA69908 in disease resistance. Biochem Biophys Res Commun. 634:203-210.

Meng, X., Jin, W., Wu, F. (2020) Novel tomato miRNA miR1001 initiates cross-species regulation to suppress the conidiospore germination and infection virulence of Botrytis cinerea in vitro. Gene. 759:145002.

Zhao, W., Li, Z., Fan, J., Hu, C., Yang, R., Qi, X., Chen, H., Zhao, F., Wang, S. (2015) Identification of jasmonic acid-associated microRNAs and characterization of the regulatory roles of the miR319/TCP4 module under root-knot nematode stress in tomato. J Exp Bot. 66(15):4653-67.

Yang, F., Zhao, D., Fan, H., Zhu, X., Wang, Y., Liu, X., Duan, Y., Xuan, Y., Chen, L. (2020) Functional Analysis of Long Non-Coding RNAs Reveal Their Novel Roles in Biocontrol of Bacteria-Induced Tomato Resistance to Meloidogyne incognita. Int J Mol Sci. 21(3):911.

Wang, J., Yang, Y., Jin, L., Ling, X., Liu, T., Chen, T., Ji, Y., Yu, W., Zhang, B. (2018b) Re-analysis of long non-coding RNAs and prediction of circRNAs reveal their novel roles in susceptible tomato following TYLCV infection. BMC Plant Biol. 18(1):104.

Wang, J., Yu, W., Yang, Y., Li, X., Chen, T., Liu, T., Ma, N., Yang, X., Liu, R., Zhang, B. (2015) Genome-wide analysis of tomato long non-coding RNAs and identification as endogenous target mimic for microRNA in response to TYLCV infection. Sci Rep. 5:16946.

Suzuki, T., Ikeda, S., Kasai, A., Taneda, A., Fujibayashi, M., Sugawara, K., Okuta, M., Maeda, H., Sano, T. (2019) RNAi-Mediated Down-Regulation of Dicer-Like 2 and 4 Changes the Response of 'Moneymaker' Tomato to Potato Spindle Tuber Viroid Infection from Tolerance to Lethal Systemic Necrosis, Accompanied by Up-Regulation of miR398, 398a-3p and Production of Excessive Amount of Reactive Oxygen Species. Viruses. 11(4):344.

Prasad, A., Sharma, N., Chirom, O. et al. (2022) The sly-miR166-SlyHB module acts as a susceptibility factor during ToLCNDV infection. Theor Appl Genet 135, 233–242.

Dey, S., Sarkar, A., Chowdhury, S., Singh, R., Mukherjee, A., Ghosh, Z., Kundu, P. (2022) Heightened miR6024-NLR interactions facilitate necrotrophic pathogenesis in tomato. Plant Mol Biol. 109(6):717-739.
